# Periprocedural safety and outcome after pump implantation for intravenous treprostinil administration in patients with pulmonary arterial hypertension

**DOI:** 10.1186/s12890-021-01541-3

**Published:** 2021-05-15

**Authors:** Jan C. Kamp, Jan Fuge, Jan F. Karsten, Stefan Rümke, Marius M. Hoeper, Da-Hee Park, Christian Kühn, Karen M. Olsson

**Affiliations:** 1 Department of Respiratory Medicine, Hannover Medical School, Carl-Neuberg-Str. 1, 30625 Hannover, Germany; 2 Department of Anesthesiology and Intensive Care Medicine, Hannover Medical School, Hannover, Germany; 3 Department of Cardiothoracic, Transplantation and Vascular Surgery, Hannover Medical School, Hannover, Germany

**Keywords:** Pulmonary arterial hypertension, Pump implantation, Treprostinil

## Abstract

**Methods:**

In this retrospective observational study, we analyzed all patients with pulmonary arterial hypertension undergoing LenusPro® pump implantation between November 2013 and October 2019 at our center. Periprocedural safety was assessed by describing all complications that occurred within 28 days after surgery; complications that occurred later were described to assess long-term safety. Clinical outcomes were measured by comparison of clinical parameters and echocardiographic measurements of right ventricular function from baseline to 6-months-follow-up.

**Results:**

Fifty-four patients underwent LenusPro® pump implantation for intravenous treprostinil treatment during the investigation period. Periprocedural complications occurred in 5 patients; the only anesthesia-related complication (right heart failure with recovery after prolonged intensive care and death in the further course) occurred in the only patient who underwent general anesthesia. All other patients underwent local anesthesia with or without short-acting (analgo-) sedation. Eighteen long-term complications occurred in 15 patients, most notably pump pocket or catheter related problems. Transplant-free survival rates at 1, 2, and 3 years were 77 %, 56 %, and 48 %, respectively.

**Conclusions:**

Subcutaneous pump implantation under local anesthesia and conscious analgosedation while avoiding intubation and mechanical ventilation is feasible in patients with advanced PAH. Controlled studies are needed to determine the safest anesthetic approach for this procedure.

**Background/Objectives:**

Intravenous treprostinil treatment via a fully implantable pump is a treatment option for patients with advanced pulmonary arterial hypertension. However, there is no consensus on the preferred anesthetic approach for the implantation procedure. Primary objective was to assess periprocedural safety with particular attention to feasibility of local anesthesia and conscious analgosedation instead of general anesthesia. Long-term safety and clinical outcomes were secondary endpoints.

## Background

Pulmonary arterial hypertension (PAH) is a progressive disease of the pulmonary vasculature, leading to death from right heart failure if left untreated [1]. Current guidelines recommend a risk-oriented treatment strategy [2, 3]. For patients stratified as high risk or with insufficient response to dual oral combination therapy, parenteral prostacyclin analogue administration is an appropriate treatment option [4]. Besides epoprostenol, which has been the first targeted PAH therapy [5], treprostinil is the second approved intravenous prostacyclin analogue for the treatment of PAH [6]. The evidence on long-term efficacy and safety of treprostinil as an add-on therapy in PAH patients with an insufficient response to initial or over the course expanded oral combination therapy is limited. Randomized controlled trials addressing this clinical situation have not been performed. In 2019, a multicenter retrospective analysis by Olsson et al. showed that only 19 % of PAH patients with pre-existing oral combination therapy were able to reach a low risk profile after initiation of treprostinil as an add-on therapy [7].

The LenusPro® (Tricumed Medizintechnik GmbH, Kiel, Germany), a fully implantable pump, is approved in Europe for continuous intravenous treprostinil administration [8]. The pump can be implanted in the abdominal subcutaneous fatty tissue. It comprises a drug reservoir, covered by a silicone septum for percutaneous refilling and a central venous catheter which is tunneled to the subclavian vein. After implantation, treprostinil solution is injected into the drug reservoir under aseptic conditions and the pump mechanism provides a constant intravenous flow. Refilling intervals depend on pump size (20 or 40 ml) and flow rates and commonly range from 14 to 28 days [9].

Perioperative mortality and morbidity are high in patients with advanced PAH undergoing major surgery or general anesthesia as described in several studies [10–15]. To date there is no evidence for feasibility and periprocedural safety of LenusPro® pump implantation during local anesthesia and conscious analgosedation.

The objective of this analysis was to analyze the periprocedural and long-term safety of PAH patients undergoing surgery for LenusPro® pump implantation under local anesthesia, combined with low-dose analgosedation for intravenous treprostinil treatment. Furthermore, we describe details of the implantation procedure applied at our center and discuss their impact on periprocedural safety.

## Methods

In this retrospective monocentric study, we analyzed the periprocedural safety of LenusPro® pump implantation for intravenous treprostinil treatment in all PAH patients who underwent this procedure at our institution between November 2013 and October 2019. Moreover, we analyzed long-term safety and we described clinical outcomes in these patients by opposing clinical baseline parameters with those obtained at 6-months-follow-up. We also analyzed the frequency of occurring complications over time up to the last available follow-up. Moreover, we compared the first and the second half of patients with regards to the frequency of occurring complications and clinical outcomes. Follow-up ended on February 29th, 2020. According to German law, Institutional Review Board approval is not required for retrospective data collection. All patients gave written informed consent.

All patients fulfilled the following inclusion criteria: (1) a diagnosis of PAH according to current recommendations; (2) age ≥ 18 years, and (3) initiation of intravenous treprostinil therapy via a LenusPro® pump. Exclusion criteria comprised (1) age < 18 years, and (2) pulmonary hypertension other than PAH.

The starting point of this study was the initiation of intravenous treprostinil treatment. Baseline assessments, obtained prior to treprostinil initiation, included selected echocardiographic parameters (right ventricular end-diastolic diameter (RVEDD) and tricuspid annular plane systolic excursion (TAPSE)), hemodynamics from right heart catheterization, six minute walking distance (6MWD), World Health Organization functional class (WHO-FC), capillary blood carbon dioxide tension levels (pCO_2_), and serum levels of the N-terminal fragment of pro brain natriuretic peptide (NT-proBNP). Follow-up assessments, obtained at 6-months-follow-up, included the same parameters, with the exception of right heart catheterization results due to too few available follow-up examinations. All complications that occurred within 28 days after surgery were considered perioperative. Risk at baseline and follow-up was calculated according to current recommendations [16].

The medical indication for treprostinil treatment was based on the discretion of the physicians in charge in accordance with current treatment recommendations. Intravenous treprostinil treatment was started at a dose of 1.25 ng/kg/min via a peripheral venous catheter and gradually increased. After uptitration under in-patient conditions, evaluation of tolerance, clinical benefit and NT-proBNP, LenusPro® pump implantation was performed. After surgery, further increase of treprostinil dose was carried through every refilling procedure up to the maximum tolerated dose, additionally refilling intervals were shortened if deemed necessary.

The flow mechanism of the LENUS Pro® pump operates by pressurized gas and does not contain a battery. The driving pressure is generated during the refills. Treprostinil is injected via a silicone septum into a drug reservoir, and a gas driven bellows is generating a constant flow regulated by a capillary. All pumps have fixed individual flow rates stated by the manufacturer. Dose adjustments are made via adapting the treprostinil concentration. Flow rate variances are regularly calculated by measuring the amount of remaining drug/liquid volume at refill.

It has been reported that in long-term application of intravenous treprostinil via LENUS Pro® pump, the actual flow rate differs from the distributor-specified fixed flow rate, starting lower than the expected rate after implantation and increasing slowly but steadily with long term use [9]. It is speculated that chemical substances within the intravenous treprostinil sodium formulation slowly cause alterations within the glass capillary over a long period of time.

The pump contains a direct port to the catheter allowing access under fluoroscopy in case of an occlusion alarm. The occlusion alarm is generated by a pressure sensor connected to an acoustic alarm which requires a battery. The manufacturer guarantees functionality of this battery for 4 years [9].

Flow rate variances are documented by the refilling pharmacist and double checked in our institution to provide patient safety and facilitate adequate dose adaptations. The manufacturer (Tricumed Medizintechnik GmbH, Kiel, Germany) guarantees that the silicone membrane allows 500 correctly performed punctions of the silicone septum.

### Perioperative management

A standardized protocol for PAH patients undergoing LenusPro® pump implantation has been established at our center in order to reduce perioperative risk as much as possible. All patients underwent extensive preoperative risk assessment including transthoracic echocardiography, 6MWD, WHO-FC, capillary blood gas analysis and measurement of NT-proBNP in addition to common anesthesia-related preoperative assessments including duplex sonography of the upper body vein status.

Preoperative anesthesiological evaluation of PAH patients was performed according to current recommendations and consensus statements [17, 18] and a comprehensive cardiopulmonary assessment (i.e. history and examination, medication, functional status, 6MWD, pulmonary function, blood gas analysis, laboratory tests (incl. creatinine, GFR, NT-proBNP), electrocardiogram, chest X-ray, and echocardiography [11].

All patients were admitted to our hospital for intravenous up-titration of treprostinil via a peripherally inserted central venous catheter prior to pump implantation. Treprostinil dose was increased every 8–12 h depending on patient’s tolerability, and pump implantation surgery was performed once a sufficient individual dose was reached.

Whenever possible, surgery was conducted during concious sedation, i.e., in local anesthesia combined with low-dose analgosedation as needed. Standard monitoring includes electrocardiogram, non-invasive blood pressure measurement, pulse oximetry, respiratory rate, and approximation of end-tidal carbon dioxide (etCO_2_). During the procedure, oxygen (4–8 l/min) was applied via face mask allowing sampling of exhaled carbon dioxide during administration of supplementary oxygen (Flexicare Dual Mask™, Flexicare Medisize Germany, Siegburg, Germany). Oxygen was delivered to maintain peripheral oxygen saturation (SpO_2_) at 92–94 %. All patients received 1–2 peripheral intravenous lines (> G18). Analgosedation was achieved by continuous infusion of remifentanil (concentration: 0.5–1 mg/50ml; infusion rate 0.02–0.15 µg/kg/min) with or without co-administration of midazolam boli (0.025–0.05 mg/kg) until the end of surgery. Depth of sedation aimed for a Richmond Agitation and Sedation Score (RASS) of 0 to – 2 (“conscious sedation“). Convective temperature management system was used to achieve normothermia (TWINWARM MoeckWarmingSystem™, Moeck&Moeck GmbH, Hamburg, Germany).

Furthermore, close attention was paid to avoid circulatory dysregulation and hypothermia as well as hypoxemia, hypercapnia or pain spikes/stress as all of these can affect pulmonary hemodynamics [19, 20]. PAH therapy was continued in all patients during the perioperative period.

### Pump implantation: surgical technique

**Fig. 1:**
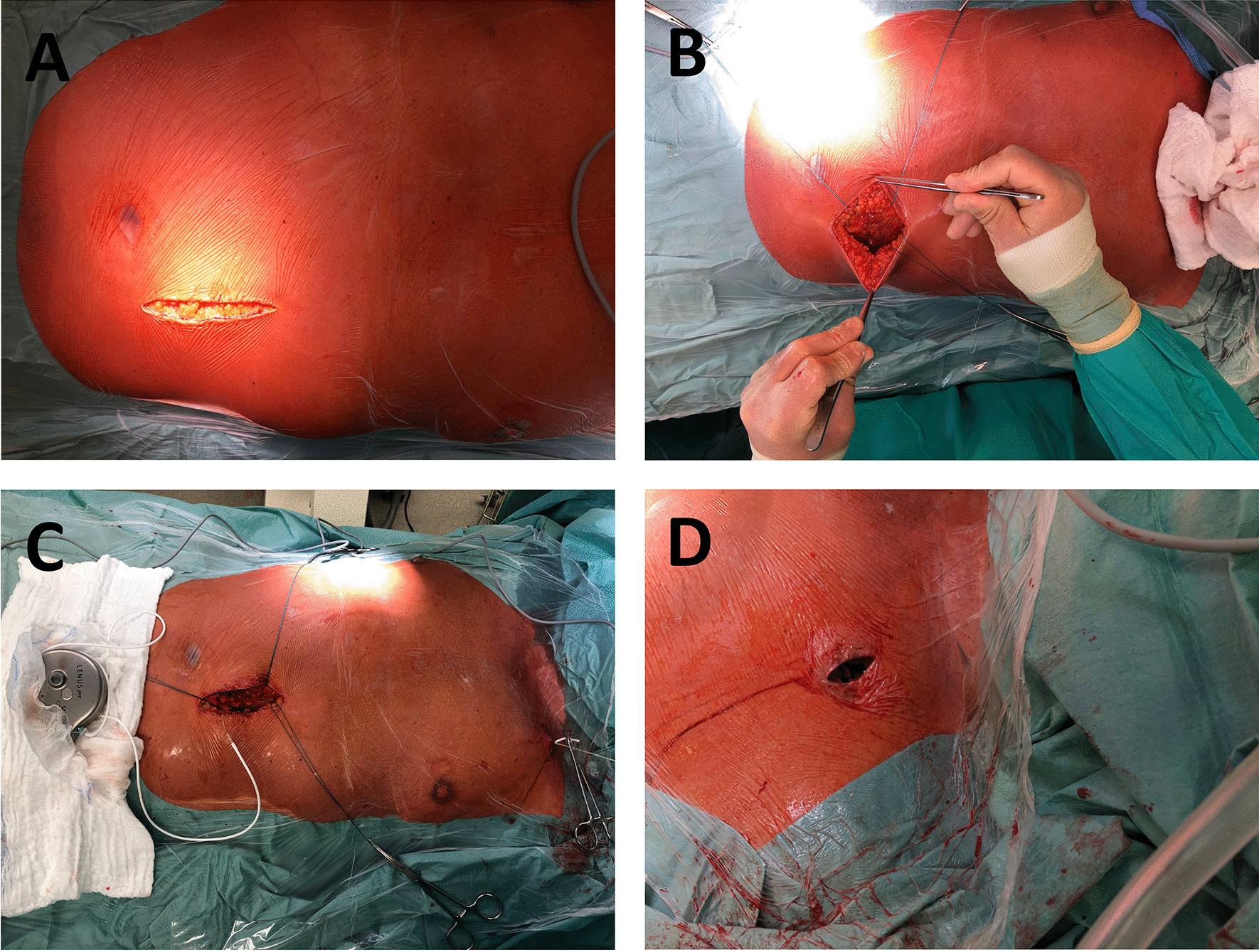
Surgical pump implantation procedure. **a** Incision in the left lower abdomen to prepare a pocket for the pump housing. **b** Subcutaneous pocket with the holding threads for the fixation of the pump. **c** Tunneling of the pump line from the abdominal incision to the subclavicular incision. **d** Subclavicular incision to puncture the subclavian vein

**Fig. 2:**
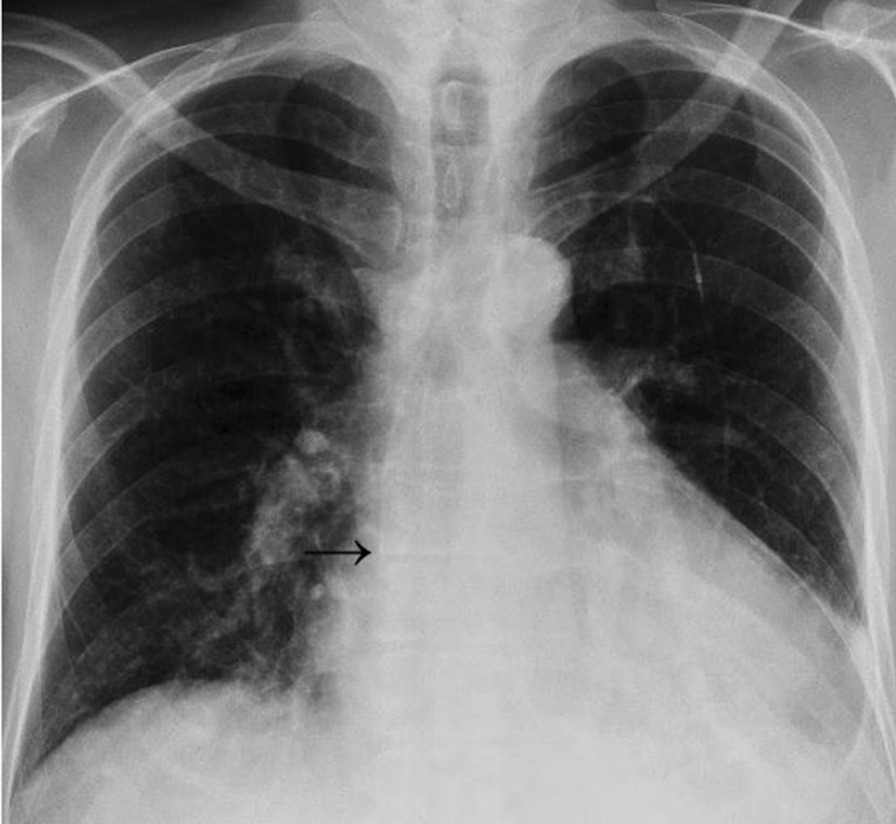
Postoperative chest X-ray. Chest fluoroscopy after surgery showing the correct localization of the catheter tip (arrow) at the superior vena cava/right atrium junction

Operations were carried out by an experienced cardiothoracic surgeon and performed in supine position in compliance with usual hygiene regulations. After extensive disinfection and sterile covering of the operating area, the target areas were subcutaneously infiltrated by local anesthetics (prilocaine 1 %, 30 ml), and intravenous antibiotics (intra-venous single-shot cefuroxime 1.5 g) were administered. After adequate exposure time, skin incision was made caudally to the left or right clavicle (Fig. 1d) with subsequent preparation of the subcutaneous adipose tissue towards the subclavian vein (left sided implantation was preferred whenever possible in order to avoid kinking of the pump line). The subclavian vein was punctured and a probe was inserted via Seldinger’s technique and moved forward fluoroscopy-guided to the superior vena cava / right atrium junction. Following this, a second skin incision was made under the left costal arch (Fig. 1a) and a subcutaneous slot was created (Fig. 1b). From there, a tunnelizer was brought through the subcutaneous tissue to the subclavian area and the pump line was pulled through the tunnel and connected to the probe using an appropriate connector (Fig. 1c). Subsequently, LenusPro® pump was moistened by vancomycin and gentamycin solution, introduced to the slot, and fixed by sutures to avoid pump rotations in the further course. Choice of pump size depended on patient’s constitution, pump pocket location, and the surgeon’s appraisal. Smaller pumps (20 ml reservoir) were used for lean patients or atypical pump positions while larger pumps (40 ml reservoir with normal or high septum) were used for all other patients. Every choice was made individually and no BMI cut-off existed. Finally, adequate position of all components was controlled by fluoroscopy (Fig. 2) followed by wound closure.

### Pump explantation in patients undergoing lung transplantation

In patients undergoing lung transplantation, right lung is always transplanted before left lung. After right-sided transplantation is completed, the surgeon makes a left-sided thoracical incision and prepares towards the left internal thoracic artery. During this procedure, the pump line becomes visible and the surgeon attaches a clamp to interrupt treprostinil administration. Pump and pump line are removed at the end of the procedure.

### Filling procedures

The first filling procedure was performed intraoperatively to avoid punction of the freshly operated site. In-hospital pump refills were performed by an experienced physician. Outpatients received refills at home by a trained nurse.

Under aseptic conditions, localization of the septum was usually done manually with support by a special localization device or ultrasound, if necessary. Correct position was verified by reflux of fluid from the pump and by checking reflux in the syringe during injection. After application of treprostinil, NaCl was injected to avoid local reactions resulting from residual amounts of treprostinil in/on the needle.

### Statistical analysis

Given that our data was observational, retrospective and post-hoc, our results were depicted in a descriptive manner without formal statistical analyses.

## Results

A total of 54 patients received an implantable pump and were included in this analysis. The patient characteristics are shown in Table 1. Two-thirds of the patients were female and median age was 46 (Q1–Q3, 35–60) years. All patients were pre-treated with PAH drugs other than treprostinil, and all patients had a follow-up of at least 6-months. N = 18 patients (33 %) had right heart catheterization approximately 1–2 weeks before pump implantation. In the remaining patients, disease progress was determined by clinical, functional, echocardiographic and laboratory parameters.

**Table 1:** Patient characteristics prior to initiation of intravenous treprostinil treatment

Result–Median (Q1–Q3) or n (%)	n = 54
Diagnosis – n (%)	
IPAH/HPAH	38 (70 %)
PAH-CTD	7 (13 %)
PAH-CHD	6 (11 %)
Others*	3 (6 %)
Age at Diagnosis (years)	42 (28–53)
Time from diagnosis to pump implantation (years)	5 (1–10)
Sex – n (%)	
Female	37 (69 %)
Male	17 (32 %)
BMI	25 (22–28)
Age at implantation (years)	46 (35–60)
Therapy	
Therapeutic anticoagulation – n (%)	27 (50 %)
LTOT – n (%)	9 (17 %)
ERA – n (%)	49 (91 %)
PDE-5-inhibitor – n (%)	40 (74 %)
Riociguat – n (%)	13 (24 %)
Iloprost i.v. – n (%)	4 (7 %)
Iloprost inhaled – n (%)	3 (6 %)
Treprostinil s.c. – n (%)	0 (0 %)
Beraprost – n (%)	1 (2 %)
Selexipag – n (%)	5 (9 %)
Right heart catheter at diagnosis (n = 54)	
Right atrial pressure (mmHg)	9 (6–14)
PAP mean (mmHg)	57 (47–67)
PAWP (mmHg)	9 (8–11)
CO (l/min)	3.4 (3.0-4.3)
CI (l/min/m^2^)	2.0 (1.6–2.5)
PVR (dyn*sec*cm^5^)	961 (739–1311)
SVI (ml/m^2^)	25 (21–32)
Mixed venous oxygen saturation (%)	63 (57–70)

Continuous variables are stated as median and interquartiles and categorical variables are stated as n and percent, unless stated otherwise

The details of the implantation procedures are shown in Table 2. The same senior cardiothoracic surgeon performed all pump implantation procedures supported by a pool of experienced cardiothoracic anesthesiologists. The conscious sedation approach allowed for a good surgical setting with patients being well sedated, not agitated and not moving. Neither vasopressor support, high flow nasal cannula (HFNC) oxygen therapy nor inhaled prostacyclin administration were needed during any procedure.

**Table 2:** Procedure details

Length of hospital stay (d)	12 (10–15)
Length of post-OP hospital stay (d)	5 (3–8)
Duration of surgery (min)	49 (42–56)
Mode of anesthesia – n (%)	
Endotracheal anesthesia	1 (2 %)
LA + analgosedation	29 (54 %)
LA + analgesia	11 (20 %)
LA + sedation	13 (24 %)
Intraop. fluid administration (ml)	532 (507–621)
Local anaesthetic (prilocaine 1 %) volume (ml) – Median (IQR)	30 (30–30)
Complications perioperative up to 28 d postop.	5 (9 %)
Complications later than 28 d	16 (30 %)

Continuous variables are stated as median and interquartiles and categorical variables are stated as n and percent, unless stated otherwise

Median duration of surgery was 49 (42–56) min. Median length of hospital stay was 12 (10–15) days mostly owing to treprostinil uptitration (the post-OP hospital stay was 5 (3–8) days). All but one patient received local anesthesia combined with analgesia, sedation, or low-dose analgosedation. One patient underwent endotracheal intubation per decision of the anesthesiologist because of preexisting moderate bronchial obstruction.

Perioperative complications occurred in 5 patients (9 %). Four of these patients suffered from surgical complications (pneumothorax, n = 1; hematoma requiring surgical revision, n = 2; and uncomplicated seroma, n = 1) and one patient suffered from an anesthesia-related complication: approximately 2 h after the end of the operation, hemodynamic instability due to right-sided heart failure occurred requiring awake veno-arterial ECMO support and a 26 days lasting stay on the intensive care unit (ICU). The patient initially recovered but eventually died 6 months after pump implantation.

Eighteen complications occurred later than 28 days after surgery in 15 (28 %) patients. The most common long-term complications were infections (4), catheter failures (6), and catheter displacements (3), imminent (2) or manifest (1) skin penetrations by the pump, hematoma (1), and pump torsion (1). Most complications occurred during the first year after pump implantation. Only two catheter failures occurred later. Replacement of the complete pump system was necessary in four patients, in one case because of a skin penetration by the catheter, in two cases because of infection, and in one case because of missing therapeutic success. The frequency of complications did not differ between the first and the last half of patients.

Smaller pumps (20 ml) were implanted in thirteen patients (24 %) while 41 patients (76 %) received larger pumps (40 ml). Complication rates did not differ between the 20 ml and the 40 ml pumps (8 % versus 10 % within the first 28 days and 31 % versus 24 % within the first year, respectively).

During the observation period, 15 patients (28 %) underwent lung transplantation and 15 patients (28 %) died. Transplant-free survival rates at 1, 2, and 3 years were 77 %, 56 %, and 48 %, respectively.

Clinical and functional outcome parameters as well as echocardiographic measurements of right ventricular function at baseline and 6-months follow up are shown in Table 3. Treprostinil dosage at time of pump implantation was 15 (range, 11–21) ng/kg/min, and the dosage at 6 months was 29 (range, 20–35) ng/kg/min.

**Table 3:** Clinical outcome after pump implantation

	Baseline (n = 54)	6-months-follow-up (n = 53*)
Lung-transplanted - n (%)	–	4
Deceased – n (%)	–	1
Right ventricular end-diastolic diameter 4 chamber view (mm)	56.6 ± 8.0	53.2 ± 8.4
Tricuspid annular plane systolic excursion (mm)	17 ± 4.6	17 ± 3.7
WHO-FC – n (%)		
Class 1	0 (0 %)	0 (0 %)
Class 2	4 (7 %)	13 (30 %)
Class 3	42 (78 %)	30 (63 %)
Class 4	8 (15 %)	5 (12 %)
6 min walking distance (m)	345 ± 154	383 ± 142
NT-proBNP (ng/l)	2986 ± 2630	3164 ± 3889
Risk Score – n (%)**		
Low	4 (8 %)	13 (28 %)
Intermediate	42 (78 %)	30 (65 %)
High	8 (15 %)	3 (7 %)

Continuous variables are presented as mean and standard deviation and categorical variables are presented as n and percent, unless stated otherwise

*1 patient had no routine visit at 6 months (+-1 month)

**Assessed by the Swedish/COMPERA method

During the observation period, 1770 filling procedures were performed and no severe complications occurred. Very few typical treprostinil associated mild local skin reactions without need for intervention were documented.

## Discussion

The present analysis was primarily performed to determine the feasibility and periprocedural safety of LenusPro® pump implantation during local anesthesia and conscious analgosedation. Secondary objectives were long-term safety of the implantable pump and long-term outcomes with intravenous treprostinil. Our data show that the procedure was generally safe and well tolerated. The only major complication occurred in a patient in whom general anesthesia had been used. The rate of surgical complications was low (9 %) and the majority of late complications (> 28 days after implantation) were associated with catheter dysfunction.

The perioperative treatment of PAH patients is challenging and depends on the successful implementation of interdisciplinary approaches (pulmonology, cardiology, surgery, anesthesiology). The anesthesiological care of PAH patients requires reasonable preoperative risk stratification (patient-related/surgery-related perioperative risks) and planning of anesthesia but also (interdisciplinary) responsibility for suitable postoperative care.

PAH patients are at elevated risk of perioperative hemodynamic deterioration and fatal complications during and after anesthesia [12]. To date, there is no consensus concerning the preferred mode of anesthesia. Current guidelines recommend the use of epidural rather than general anesthesia whenever possible [2] but this procedure can cause fluid shift which is challenging for the fragile circulation of PAH patients. As a consequence, we established a standardized implantation procedure at our center, using solely low dose analgosedation and local anesthesia.

We preferred the administration of short acting drugs such as remifentanil with or without co-administration of midazolam. Remifentanil is easily titratable to achieve optimal analgesia. It has a short, stable context-sensitive half-time, and wears off quickly and predictably after stopping the infusion. Midazolam is often co-administered with opioids and provides reliable procedural (analgo-) sedation, anxiolysis, and amnesia, but is less titratable than remifentanil. In cases of loss of spontaneous ventilation or airway reflexes adverse effects can be antagonized with an effective antagonist. We avoid the administration of propofol although it has accounted for its popularity in the field of procedural sedation. Propofol (especially following bolus administration) has significant cardio-respiratory depressant effects, which may be exacerbated in combination with other agents (e.g., opioids), and may result in detrimental effects in patients with PAH [21].

In our study, the only anesthesia-related complication, i.e. right-sided heart failure requiring ECMO support, occurred in the patient who underwent general anesthesia and endotracheal intubation. Although single cases should not be overemphasized, this may serve as reminder of the fragility of patients with advanced PAH and reinforce the concept of avoiding general anesthesia in this patient population whenever possible.

There are few data on safety of LenusPro® pump implantation procedures. Previous studies primarily addressed long-term efficacy and safety of intravenous treprostinil treatment in PAH patients rather than the influence of different anesthetic approaches and the surgical implantation procedure on perioperative safety [8, 22–24]. One study addressed the implantation procedure itself using a SynchroMed® II drug pump, summarizing surgical techniques and complications [25]. Procedure-related complications were reported in 17 % of the patients within the first 28 days of the procedure, but anesthesia-related outcomes were not reported. The majority of complications that occurred in the above-mentioned studies were pump pocket related complications like hematoma or seroma, catheter-related problems like dislocation or skin penetration, and, although substantially less frequent, pump related complications like flow rate variances or technical defects. Both conscious sedation and general anesthesia were used and no anesthesia-related complications were reported with any modality.

Based on the results of these studies, no strong recommendation on the preferable anesthetic approach for LenusPro® pump implantation can be made. Mortality and morbidity are considerable in patients with severe PAH regardless of the choice of anesthesia. So far, there is no clear advantage of local anesthesia and (analgo-) sedation over general anesthesia. Still, the present study shows that pump implantation without general anesthesia is feasible and we believe that this approach should be chosen whenever possible. It is important, however, to note that our data were derived from a tertiary care center with large PH, pulmonary endartectomy, lung transplant, and awake extracorporeal membrane oxygenation program [26], where the anesthesiologists have substantial expertise with the perioperative management of patients with advanced PH.

Our study has several limitations including the retrospective design, the lack of a control group, the small sample size, and the single center setting.

## Conclusions

In conclusion, subcutaneous pump implantation under local anesthesia and conscious sedation while avoiding intubation and mechanical ventilation is feasible in patients with advanced PAH. Controlled studies are needed to determine the safest anesthetic approach for this procedure.

## Data Availability

Data are available on reasonable request to corresponding author.

## Abbreviations

etCO2: End-tidal carbon dioxide tension levels
PAH: Pulmonary arterial hypertension
6MWD: Six minute walking distance
WHO-FC: World Health Organization functional class
pCO2: Capillary blood carbon dioxide tension levels
NT-proBNP: Serum levels of the N-terminal fragment of pro brain natriuretic peptide
RASS: Richmond Agitation and Sedation Score
RVEDD: Right ventricular end-diastolic diameter
SpO2: Peripheral oxygen saturation
TAPSE: Tricuspid annular plane systolic excursion
HFNC: High flow nasal cannula
